# Examination of daily abusive supervision effects on next-day employee wellbeing: a spillover perspective

**DOI:** 10.1080/00049530.2023.2264938

**Published:** 2023-10-29

**Authors:** Yao Zhu, Chaoyue Zhao, Jin-Ying Zhuang

**Affiliations:** Shanghai Key Laboratory of Mental Health and Psychological Crisis Intervention, School of Psychology and Cognitive Science, East China Normal University, Shanghai, China

**Keywords:** Daily abusive supervision, insomnia, daily wellbeing, rumination

## Abstract

**Objective:**

Most previous studies on abusive supervision (AS) and employee wellbeing have used cross-sectional designs and explored long-term effects. However, AS has been reported to vary on a daily basis and this variance exceeds inter-person variance. Therefore, the current study examined the short-term (within 1 day) effects of leaders’ daily AS on employee sleep and wellbeing. Based on the spillover effect theory, we posited that daily AS is a negative experience that can lead to insomnia, thereby affecting next-day wellbeing. In addition, we hypothesized that these effects could be moderated by individuals’ tendencies to engage in rumination (low or high).

**Method:**

A daily diary design was used to examine the relationship between daily AS and next-day wellbeing. Our analysis of data from 128 full-time employees across 10 consecutive working days using multi-level model.

**Results:**

Our results showed that insomnia mediated the relationship between daily AS and next-day employee wellbeing, and further showed that this relationship was moderated by rumination.

**Conclusions:**

These data demonstrate a spillover effect from AS at work to quality of sleep at home, and that use of rumination as a coping strategy can exacerbate the effects of AS on insomnia and next-day employee wellbeing.

## Key Points

**What is already known about this topic**:

1. AS, as a negative leadership style, exerts detrimental effects on employees’ well-being.
2. AS has a spillover effect that extends its negative effects into employees’ home-domain.
3. Rumination, as a maladaptive coping mechanism, exacerbates the deleterious effects of negative stressors on psychological well-being.

**What this topic adds**:

1. In contrast to the conventional perspective that AS is a form of leadership, we conceptualise it as a type of negative daily behaviour and investigate its spillover effect on employees’ wellbeing the following day.
2. Through the experience sampling method, we can not only identify the directional relationship between AS and employees’ well-being but also capture the temporal spillover effects of AS.
3. We further investigated the internal mechanisms and boundary conditions of daily AS on employee well-being, revealing that rumination exacerbates the spillover effects of such behaviour.

Abusive supervision (AS) is defined in terms of subordinates’ perceptions of supervisors engaging in sustained excessive hostile verbal and nonverbal behaviours, excluding physical contact (Tepper, 2000). The negative effects of AS on employees have been widely discussed. AS has been shown to be a negative workplace stressor that can impede employee performance, reduce employee wellbeing over time, and even contribute to the development of symptoms of depression and anxiety in employees (Tepper et al., 2017).

In the literature, AS has been characterised as a stable style of leadership, both conceptually and methodologically (Lin et al., 2013; Tepper, 2000). Conceptually, it has been considered to be a continuous, sustained behaviour against employees (Tepper, 2000). Methodologically, cross-sectional designs have typically been used to explore the antecedents and consequences of AS. However, based on findings of substantial intra-individual variation in AS, researchers have begun to focus on daily AS behaviours rather than abusive leaders (Barnes et al., 2015). Leaders’ interactions with employees may vary day to day depending on the current work environment and events. The topic of employee wellbeing has garnered increasing attention from both corporations and scholars alike. With the recognition that wellbeing experiences daily fluctuations, researchers have redirected their focus towards short-term rather than long-term wellbeing. The association between AS and employee wellbeing has been extensively validated (eg., Lin et al., 2013; Morsch et al., 2020); however, limited scholarly attention has been devoted to investigating leaders’ daily behaviours in terms of AS and their immediate impact on wellbeing. Therefore, in this study, AS is examined in terms of a leader’s daily behaviour and its short-term impact on employee wellbeing by analysing the relationship between AS one day, treated as an independent variable, and employees’ next-day wellbeing, treated as a dependent variable.

Spillover effects are affective experiences or events that transmit from one domain (e.g., work) to another (e.g., home) (Bolger et al., 1989). Emotionally taxing experiences, including AS, can extend beyond working hours, causing employees to experience ongoing stress outside of the workplace (Tu et al., 2018). Perseverative mental replay of AS episodes after work can elevate levels of alertness, and this spillover effect may undermine night-time sleep quality. Importantly, it is well known that a lack of restful sleep degrades physical and mental health, and sleep has been shown to be closely related to wellbeing (Hamilton et al., 2007; Simione et al., 2020). Therefore, the present study examines the hypothesis that night-time insomnia is a mediating mechanism that transmits the effects of AS to employees’ next-day wellbeing.

Previous studies have suggested that individuals have relatively stable cognitive, affective, and behavioural patterns in response to certain types of situations (Yang & Diefendorff, 2009). According to this view, different individuals may react differently when faced with AS depending on their stable response predispositions. Notably, rumination, which is generally regarded as a negative way of coping with events, has been shown to exacerbate the negative effects of stressful events (Zhang et al., 2017). Thus, in the current study, we included rumination as a between-individual moderating variable and predicted that a tendency to engage in rumination would exacerbate AS-related insomnia. This analysis will be helpful for determining potential boundary conditions of the spillover effects of AS.

The work presented here makes three specific novel contributions to the literature. First, we explore short-term consequences of AS and report our findings in relation to AS behaviour by leaders acting as a negative stressor that can affect employees’ next-day wellbeing and sleep within 24 hours. This analysis expands the consequence variables of AS to some extent.

Second, this study enriches the literature on the spillover effects of daily AS. Most studies on AS spillover effects in the literature have a cross-sectional design (Tu et al., 2018; Wu et al., 2012), which is not well-suited for detecting daily changes related to exposure to AS or for the effective capture of spillover effects. Here, a daily diary design that allows the capture of spillover effects of AS that lead to lower quality night-time sleep immediately following the experience was adopted instead.

Third, we identify rumination as a trait tendency that exacerbates the spillover effects of AS. This result underscores the maladaptive consequences of rumination as a coping strategy for negative experiences and the potential for rumination to amplify the impact of negative events on individuals’ wellbeing.

## Theoretical background and hypotheses

### Daily as and next-day wellbeing

AS is constituted by prolonged emotional or psychological mistreatment of subordinates, including the ridiculing of subordinates in front of others, withholding important information, and the use of disparaging language, threats, and intimidation tactics (Lin et al., 2013; Zellars et al., 2002). There is a substantial history of research on AS and wellbeing-related variables. From such research, it has been established that AS may lead to lower subjective wellbeing, heightened psychological pressure, and heightened depression and anxiety in employees (Tepper et al., 2007, 2017).

Previous research has emphasised the sustained nature of AS and has tended to view it as a relatively stable form of leadership, both conceptually and methodologically (Barnes et al., 2015). According to this perspective, AS is a form of abusive leadership – a concept that describes that tendency of some leaders to use abusive tactics in the management of their employees, with recognition of a spectrum of severe and moderate manifestations (Liao et al., 2021). Conversely, though there is inter-individual variation in AS related to individual leadership traits and management styles, AS behaviour has been shown to be discrete and episodic, and hence temporally dynamic (Beal et al., 2005; Liao et al., 2021). Based on such observations, it has been hypothesised that particular daily work events faced by supervisors and their moods will determine whether they are likely to use AS techniques on any given day (Qin et al., 2020). According to this perspective, AS can be analysed as a behaviour that fluctuates daily rather than as a persistent leadership style. On the basis of the hypothesis that AS encompasses discrete behaviours, the expression of which is variable, Johnson et al. (2012) found that abusive supervisory behaviour varied more within supervisors than it did between supervisors. Subsequently, Barnes et al. (2015) developed the concept of daily AS and shifted their focus of study from abusive leadership to abusive behaviour. They found that leaders’ daily sleep quality affected their own abusive supervisory behaviour (Barnes et al., 2015), thus demonstrating that AS is dynamic and can change in response to factors that fluctuate, such as mood, self-control, salient goals, and activated identities (Barnes et al., 2011; Dalal et al., 2009).

Working from the precept that AS can be analysed as a daily behaviour, we can reconsider its consequences for employees. In previous research wherein AS has been treated as an ongoing behaviour or a stable type of leadership, long-term and cumulative consequences of ongoing AS have been explored. For example, Lin et al. (2013) described AS an interpersonal stressor with negative consequences for subordinates, such as poor mental health and job dissatisfaction. However, if AS is a set of behaviours that changes on a daily basis, then episodes of heightened AS intensity may have immediate consequences for employees. Thus, we set out to explore whether such episodes have short-term negative impacts on employees.

Among the many variables that could be influenced by AS, we focused our attention on employee wellbeing, a broadly structured comprehensive outcome. According to Zheng et al. (2015) definition, employee wellbeing is not only the cognisance and perception of an employee’s satisfaction, but also employees’ emotional experience and satisfaction in work and non-work domains. It includes three dimensions: life, workplace, and psychological wellbeing (Zheng et al., 2015). Based on this definition, employee wellbeing is a broadly representative dependent variable that is well-suited to reveal the negative effects of AS. Furthermore, there has been a convergence of findings from recent studies that have explored wellbeing at the within-individual level demonstrating that wellbeing is a dynamic variable that can fluctuate day to day (Bakker et al., 2019; Cangiano et al., 2019; Möwisch et al., 2019). Therefore, previous cross-sectional studies have primarily focused on the chronic impact of AS on wellbeing over time. However, our research aims to investigate the immediate effects of AS and specifically examine employees’ psychological well-being at a daily level. Thus, the purpose of this study was to explore whether daily AS affects employees’ daily wellbeing.

The cross-sectional design adopted in most previous studies examining AS and wellbeing (e.g., Bowling & Michel, 2011; Lin et al., 2013) is flawed in that it neither identifies relationship directions nor captures immediate effects of AS. This study treats AS as a daily behaviour, in accordance with the work of Barnes et al. (2015), and posits that it creates an immediate sense of stress for employees, thus affecting their wellbeing the next day. We thus hypothesised the following:

*Hypothesis 1*: Daily AS is negatively related to next-day employee wellbeing.

### The mediating role of daily insomnia

Spillover theory is based on the precept that one’s work experiences and family life are interrelated (Lee et al., 2018). It provides a holistic view wherein it is reckoned that life events and experiences in different domains affect one another (Ilies et al., 2009). Negative events in the workplace may spill over into the home domain, leading to rumination and elevated levels of alertness that, eventually, degrade sleep quality (Tariq et al., 2020; Wagner et al., 2014; Wang et al., 2013). Therefore, AS, as a negative interpersonal stressor, has the potential to produce pressure outside the workplace, resulting in insomnia (Tariq et al., 2020). Cross-sectional design studies (e.g., Han et al., 2017; Yuan et al., 2020) are not suited to detecting dynamic short-term spillover effects, wherein insomnia during a particular night may follow stressful events experienced by employees during the preceding day. Therefore, this study adopts a within-individual design to capture acute spillover effects.

Insomnia, defined as difficulty with falling and/or staying asleep (Jenkins et al., 1988), has been estimated to afflict up to 50% of the general population depending on the criteria applied (for review, see Riemann et al., 2010). Reported negative impacts of insomnia on employees in the workplace include the development of burnout, reduced productivity, and reduced wellbeing (Bolge et al., 2009; Espie et al., 2018; Jansson-Fröjmark & Lindblom, 2010). The inverse correlation between insomnia and wellbeing is widely acknowledged. Insomnia, being a condition characterised by sleep deprivation, has been reported to reduce both short- and long-term wellbeing (Karlson et al., 2013; Simione et al., 2020). Sleep is regarded as a homoeostatic process required to restore cognitive capacity (e.g., attention) and strength (e.g., energy) (Barnes & Hollenbeck, 2009). Consistent with these important biological roles, lack of sleep reduces psychological wellbeing and, in the context of employee considerations, may thus impede the ability of employees to regain their energy and strength to healthy levels that enable them to cope with challenges, thus leading to reduced wellbeing across the home and work domains of life. Therefore, based on the inference that employees’ insomnia may affect their next day’s wellbeing, we propose the following hypothesis:

*Hypothesis 2*: Daily insomnia mediates the relationship of daily AS with next-day wellbeing.

### A moderating role of rumination

Rumination is a behaviour that has been defined as “prolonged cognitive activation of stressors already experienced” (Frone, 2015, p. 33); and the tendency to engage in rumination in response to stressors can be viewed as an individual characteristic (Zhang et al., 2017). The tendency to employ rumination as a coping mechanism has been described as a personality trait wherein deliberation is triggered by exposure to stressors (Ciarocco et al., 2010; Key et al., 2008; Watkins & Roberts, 2020). Generally, rumination is considered to be a maladaptive coping strategy (Michl et al., 2013) due to perseverative thoughts prolonging stressors’ negative effects on the ruminating person (Watkins & Roberts, 2020). Negative consequences of rumination include hesitant behavioural responses and emotions such as depression and anxiety (Zhang et al., 2017).

It is well documented that rumination can exacerbate the spillover effects of workplace stressors. For example, Zhang et al. (2017) found that individuals with a ruminative predisposition experience higher levels of work-family conflict after suffering workplace ostracism. Donahue et al. (2012) argued that individuals who engage in high levels of rumination are prone to repetitive thinking about work-related matters at home, even though doing so is unproductive and draining. Therefore, we deduce that high-rumination individuals may, after being subjected to AS, think repeatedly about such interactions and engage in self-blame regarding the causes of such incidents. This repeated thinking about negative events can continue during the night at home, thus exacerbating night-time insomnia (Yuan et al., 2018). On the contrary, low-rumination individuals will not ruminate substantially on the experiences and causes of AS, and thus should not be subjected to spillover effects of AS into the home domain. Here, we include rumination as a person-level moderator. Considering a moderating effect of rumination together with the indirect effect from Hypothesis 2, we posit that indirect effects from daily AS to next-day wellbeing via insomnia will be especially strong for individuals with high levels of rumination.

*Hypothesis 3*: Rumination moderates the relationship between daily AS and insomnia such that this relationship is stronger for high-rumination individuals than for low-rumination individuals.

*Hypothesis 4*: Rumination moderates the indirect effect in Hypothesis 2 such that it is stronger for high-rumination individuals than for low-rumination individuals.

**Figure 1.**
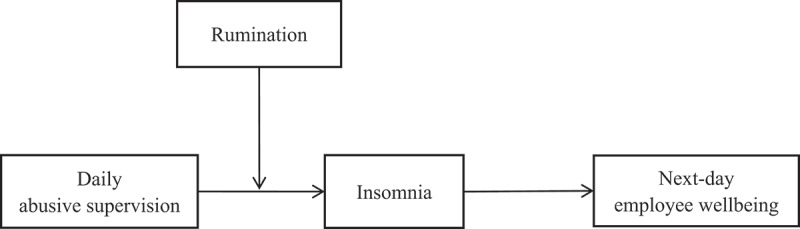
Theoretical model.

A theoretical model encompassing the presently hypothesised relationships is presented in Figure 1.

## Method

### Participants

Approval from the institutional review board of the East China Normal University (HR1–1060–2020) was obtained prior to enrolling participants in this study. We collected data from a total of 156 participants recruited through word-of-mouth and research recruitment ads in China. These recruitment methods, which have been used successfully in prior diary-based research (Butts et al., 2015), enabled a diverse pool of potential participants to be contacted. The inclusion criteria were being 18 years or older, working full-time during normal business hours (i.e., not working night shifts), and having a work history of at least 3 months in one’s current jobs. We invited potential participants to read a consent letter that provides complete information about the study, after which they could decide to participate or not.

Twenty-eight participants did not have at least three observation days and were therefore excluded. Thus, the final study sample consisted of 128 participants, of which 54 (42.2%) were female. The cohort had a mean age (±standard deviation, SD) of 37.07 (±9.72) years and a mean organisational tenure of 15.09 (±11.11) years. Participants each received 35 yuan in compensation.

### Procedure

Data were collected in two phases. In the initial phase, participants completed a baseline survey encompassing inquiries regarding their demographics, including gender, age, and occupation. Additionally, they provided their baseline measurements of rumination, AS, insomnia, and wellbeing. Commencing a week later, in the second phase, participants completed twice-daily surveys for 10 working days (i.e., Monday-Friday for two weeks). We adopted an interval-contingent design such that there was a specific time window during which participants were expected to complete their daily surveys (Fisher & To, 2012). The research team sent out an email at 7:00 a.m. with a link to the morning survey, which consisted of questions about participants’ sleep the night before, and asked participants to complete the survey within the next three hours. Those who had not completed the survey received a 9:00 a.m. reminder. The morning survey was closed at 10:00 a.m. At 5:00 p.m., participants received their evening survey email, which asked them to complete that survey after work. The evening survey consisted of questions about AS, wellbeing, and workload during the workday. Around 8:00 p.m., a reminder was sent to those who had not completed the evening survey yet. The evening survey was closed at 11:00 p.m. All surveys were administered in Chinese. We followed Brislin’s (1980) translation-back translation procedure to ensure the accuracy of translation. Simultaneously, we made adjustments to the scale items in order to enhance its suitability for our daily diary research approach. Specifically, for the evening measurements of AS and employee wellbeing scales, we incorporated “today” before each item; whereas for the morning measurements of the insomnia scale, we included “last night” before each item.

Our study hypotheses involve the independent variables of AS and workload on one day, Day t, and the dependent variables of night-time insomnia and wellbeing, surveyed on Day t + 1. Insomnia and wellbeing were also assessed on Day t to serve as control variables. Therefore, a complete daily observation required that the participant took both the morning and evening surveys on Day t and Day t + 1. We received 972 of 1248 possible daily observations (156 people × 8 days), resulting in an overall participation rate of 77.88%.

### Measures

Participants answered all survey items on a 5-point Likert scale from 1 (strongly disagree) to 5 (strongly agree).

#### Abusive supervision (day t evening survey)

AS was assessed with a five-item scale developed by Tepper (2000). We adjusted the time frame so that the items referred specifically to Day t. An example item is “Today, my supervisor told me that ‘your thoughts and feelings are stupid’”. The mean Cronbach’s alpha value for this scale was .97 (±.01) (range = .96–.98).

#### Insomnia (day t + 1 morning survey)

Insomnia was assessed in the Day t + 1 morning survey with a four-item sleep quality scale produced by Jenkins et al. (1988). An example item is “Last night, I had trouble falling asleep”. The mean Cronbach’s alpha value for this scale was .90 (±.03) (range = .84–.92).

#### Wellbeing (day t + 1 evening survey)

In the Day t + 1 evening survey, participants were asked to complete the employee wellbeing scale developed by Zheng et al. (2015). To reduce participant burden, we selected three items from each of the three subscales of the wellbeing scale: life wellbeing (e.g., “Today, my life is very fun”), workplace wellbeing (e.g., “Today, I could consistently find ways to enrich my work”), and psychological wellbeing (e.g., “Today, I feel good about myself, and I’m confident”). The mean Cronbach’s alpha value for this scale was .96 (±.01) (range = .95–.97).

#### Rumination (baseline survey)

We assessed rumination in a baseline survey with a five-item work-related worry and rumination scale (Flaxman et al., 2012). An example item is “I have been concerned about mistakes I made at work”. The Cronbach’s alpha value for this scale was .93.

#### Control variable

Considering the established association between workload and insomnia (Yang et al., 2018), we employed a single-item query to measure daily workload as an independent control variable, specifically assessing participants’ perception of their workload intensity for the day with the statement “Today, I experienced a heavy workload”.

### Analytic strategy

Means, within-subject SDs, and between-subject SDs of the study variables were determined. Intraclass correlation coefficients (ICCs), and inter-class (Pearson) correlations among the variables were calculated. Multilevel confirmatory factor analysis was conducted to examine the factor structure of study variables. These preliminary analyses were conducted in SPSS (IBM, USA).

We subjected our nested data to multilevel modelling in Mplus 8.6. Days (level 1) were nested within individuals (level 2). Three models were tested. In Model 1, we examined the effect of daily AS on next-day wellbeing while controlling for prior-day workload and wellbeing at level 1. In Model 2, we examined our mediation hypotheses by estimating the effect of AS on insomnia, while controlling for prior-day workload and insomnia, and analysed the effect of insomnia on next-day wellbeing while controlling for prior-day wellbeing. In Model 3, we added level-2 rumination to the factors included in Model 2 and we specified the cross-level effect of rumination on the random slope between AS and insomnia measurement results. To prevent the within-person level analyses from being confounded by between-person level relationships, within-person predictors were mean centred such that variable deviations were related to each individual’s own 2-week average. Between-person predictors were grand-mean centred. Model fitness was determined based on χ^2^, comparative fit index (CFI), and root mean square error of approximation (RMSEA) values. Simple slope test results are reported as γ variables and indirect effect analysis results are reported as conditional indirect effect values with 95% confidence intervals (CIs). In all cases, *p* < .05 was considered significant; otherwise, analysis results are indicated as not significant (n.s.).

## Results

### Preliminary analysis

Descriptive statistics (means and SDs) and Pearson correlations among study variables are reported in Table 1. One-way analyses of variance showed that there were significant between-person variances in insomnia [(ICC1) = .57, *F*_127, 844_ = 11.03, *p* < .001] and wellbeing (ICC1 = .58, *F*_127, 844_ = 11.23, *p* < .001), thus supporting the use of multilevel modelling for incorporation of the nested nature of the data (Bartko, 1976; James, 1982).

**Table 1.** Descriptive statistics of and correlations among study variables.

Variable	Mean	Within-person SD	Between-person SD	1	2	3	4	5
*Level 1*								
1 AS	1.95	.75	.59		.55**	−.39**	.30**	−.18**
2 Insomnia	2.41	.93	.75	.68**		−.47**	.30**	−.20**
3 Wellbeing	3.60	.82	.67	−.38**	−.55**		−.14**	.24**
4 Workload	2.75	1.23	.94	.44**	.46**	−.25*		−.27**
*Level 2*								
5 Rumination	3.16		.96	−.25**	−.27**	.32**	−.34**	

Correlations above (*N* = 972) and below (*N* = 128) the diagonal are within-person level and between-person level correlations, respectively. Each person’s daily observations were aggregated for the calculations. AS, abusive supervision. SD, standard deviation. *p < .05; **p < .01.

For our multilevel confirmatory factor analysis conducted to examine factor structure, we first fit the data to a four-factor (AS, insomnia, rumination, and wellbeing) model, in which each item was loaded on its respective latent variable. This four‐factor model fitted well with our data [χ^2^ (356) = 1134.32, *p* < .001, CFI = .91, RMSEA = .05]. Subsequently, an alternative three-factor model wherein items from AS and insomnia were loaded on one latent variable was tested. This model provided a worse fit than the four-factor model [χ^2^ (361) = 2161.74, *p* < .001, CFI = .78, RMSEA = .07]. These results support maintaining a distinction among the daily constructs.

### Hypothesis testing

Our multilevel modelling results are reported in Table 2 with unstandardised coefficients and standard errors. In Model 1, daily AS was found to affect next-day wellbeing (γ = −.29, *p* < .001), thus supporting Hypothesis 1. In Model 2, daily AS affected insomnia (γ = .25, *p* < .001), insomnia affected next‐day wellbeing (γ = −.14, *p* < .001), and an indirect effect of daily AS on next-day wellbeing via insomnia was observed (indirect effect = −.07, *p* < .05, 95% CI [−.131, −.006]), thus supporting Hypothesis 2.

**Table 2.** Multilevel modelling results.

Factor	Model 1	Model 2		Model 3	
	Wellbeing	Insomnia	Wellbeing	Insomnia	Wellbeing
*Level 1*					
Workload	.07 (.04)	.01 (.01)	–	.01 (.01)	–
Insomnia control	–	−.02 (.02)	–	−.02 (.01)	–
Wellbeing control	−.02 (.05)	–	.09 (.07)	–	.07 (.06)
AS	−.29 (.06)***	.25 (.06)***	−.20 (.05)***	.27 (.06)***	−.37 (.06)***
Insomnia	–	–	−.14 (.04)***	–	−.24 (.04)***
*Level 2*					
Rumination	–	–	–	−.00 (.00)	.20 (.06)***
AS × Rumination	–	–	–	.13 (.06)*	–

Workload data are from Day t evening survey; insomnia control data are from Day t morning survey; insomnia data are from Day t + 1 morning survey; wellbeing control data are from Day t evening survey; wellbeing data are from Day t + 1 evening survey. Between-person level sample size = 128; within-person level sample size = 972. AS, abusive supervision. *p < .05; **p < .01; ***p < .001.

**Figure 2.**
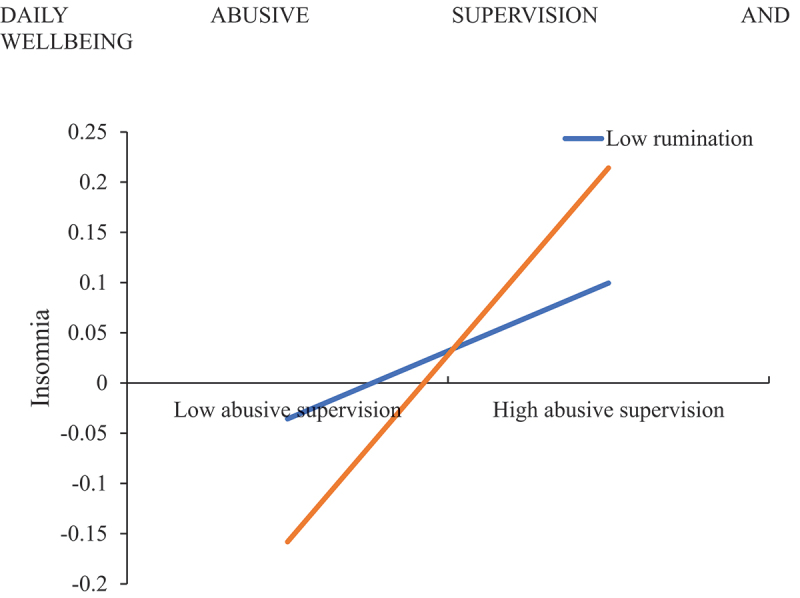
Moderating effect of individuals’ tendency to engage in rumination on the relationship between exposure to abusive supervision and insomnia.

In Model 3, there was a significant interaction between AS and rumination (γ = .13, *p* < .05), indicating that rumination moderated the relationship between AS and insomnia. The pattern (Figure 2) indicates that the positive relationship between AS and insomnia was stronger in high-rumination participants than in low-rumination participants, thus supporting Hypothesis 3.

Simple slope tests showed that the effect of AS on insomnia was significant when rumination was high (γ = .39, *p* < .001) but not when rumination was low (γ = .15, n.s.). Further, a conditional indirect effect of daily AS, via insomnia, on next-day wellbeing was significant for high-rumination participants (conditional indirect effect = −.10, *p* < .001, 95% CI [−.145, −.046]), but not for low-rumination participants (conditional indirect effect = −.04, n.s., 95% CI [−.079, .008]). The difference between these indirect effects for the two rumination-tendency groups was significant (difference = −.06, *p* < .05, 95% CI [−.117, −.003]). Thus, Hypothesis 4 was supported.

## Discussion

### Theoretical and practical implications

Our treatment of AS as a daily leadership behaviour that may have short-term effects on employee wellbeing the next day is consistent with previous research indicating that AS is dynamic within individuals, while further identifying short-term consequences of AS. Hence, the negative effects of AS on employees are not limited to cumulative effects over time, but rather may occur immediately after the experience of AS. Our findings suggest that employee wellbeing can be volatile, with employees experiencing reduced wellbeing the day after being subjected to AS. The validation of this relationship provides a more comprehensive understanding of the negative effects of AS. Notably, our use of daily diary analysis enabled us to explore the micro-level dynamics of AS without sacrificing ecological validity (Cangiano et al., 2019), thereby overcoming the challenge of AS being difficult to capture and manipulate in controlled settings.

We then verified that daily insomnia plays a mediating role between daily AS and next-day employee wellbeing consistent with spillover effect theory. Given that insomnia and wellbeing are inextricably linked (Hamilton et al., 2007), we believe that the stressful experience of AS first spills over into the employee’s home domain, leading to night-time insomnia, which in turn can lead to reduced wellbeing the next day. At the same time, our assessment of directly and indirectly related variables at different time points enables us to infer at least a likely causal direction (Larson & Csikszentmihalyi, 2014). Importantly, our daily diary analysis allowed us to capture immediate spillover effects, which supported our hypotheses.

Third, we identified a personality trait variable – namely, the tendency to engage in rumination – that augments spillover effects of AS. Building upon previous research suggesting that stressful experiences at work activate ruminative thinking (Yuan et al., 2018), we analysed rumination tendency as a between-individual variable and found that high-rumination individuals were more likely than low-rumination individuals to experience insomnia after being subjected to AS, thereby worsening their wellbeing the next day. This result enables us to identify a key boundary condition and enriches the research on spillover effects of AS.

Our findings generate a number of practical implications. Firstly, we used the experience sampling method to demonstrate that AS has an immediate impact on employee wellbeing, thus underscoring that AS can act as a short-term stressor rather than being only a chronic stressor. In practical terms, our findings, together with related literature, suggest that managers should be mindful of their words and actions, not only to not be a characteristically abusive leader, but also to not engage in episodes of abusive behaviour in their daily work lives. We recommend that managers be open and respectful to subordinates and that they work to create an equitable and harmonious organisational climate with the aim of ensuring that subordinates have a high sense of wellbeing in their work settings (Tepper et al., 2007). Secondly, we found that rumination can exacerbate the spillover effects of negative stressors in the workplace. Accordingly, it would behove managers to identify ruminant subordinates and to provide them with conscientious support. Alternatively, or in addition, managers should consider conducting psychological training within the organisation to enhance employees’ psychological capital and optimism levels and thus, potentially, lessen employees’ engagement in rumination (Roche et al., 2014).

### Limitations and future directions

Our study has several limitations. First, our analyses were based on self-reported variables, which can result in common method variance issues (Podsakoff et al., 2003) consequent to people tending to respond to questions in ways that present themselves in a favourable light (Cangiano et al., 2019; Wehrt et al., 2020). Future research could consider collecting multi-source data to obtain more objective information.

Second, our daily diary-based design involved data collection of different variables at different timepoints. Although this approach had the benefit of enabling us to analyse a delayed effect of daily AS on next‐day wellbeing via insomnia and the results obtained via this approach can be strongly indicative of causal directionality, causal conclusions cannot be made. Future studies may verify the presently suggested causal relationship between daily AS and wellbeing by employing experimental methods that manipulate employees’ perceptions of AS and by exploring the consequent effects on wellbeing.

Third, abstract analytic rumination, which focuses on the causes, consequences, and meaning of a problem, is considered maladaptive. On the other hand, constructive repetitive thinking or concrete-experiential rumination that centres around developing a plan, making decisions, and taking action to address the problem is regarded as adaptive (Watkins, 2008). Our study exclusively focuses on work-related rumination, considering it as a maladaptive coping strategy. Future research should explore the potential effects of different forms of rumination, including investigating whether positive rumination can serve as an adaptive mechanism for managing workplace stress.

## Data Availability

The data that support the findings of this study are available from the corresponding author upon reasonable request.
